# Microgravity Induction of TRAIL Expression in Preosteoclast Cells Enhances Osteoclast Differentiation

**DOI:** 10.1038/srep25143

**Published:** 2016-05-04

**Authors:** Yuvaraj Sambandam, Kelsey L. Baird, Maxwell Stroebel, Emily Kowal, Sundaravadivel Balasubramanian, Sakamuri V. Reddy

**Affiliations:** 1 Darby Children’s Research Institute, Charleston, SC, USA; 2 Radiation Oncology, Medical University of South Carolina, Charleston, SC, USA

## Abstract

Evidence indicates that astronauts experience significant bone loss in space. We previously showed that simulated microgravity (μXg) using the NASA developed rotary cell culture system (RCCS) enhanced bone resorbing osteoclast (OCL) differentiation. However, the mechanism by which μXg increases OCL formation is unclear. RANK/RANKL signaling pathway is critical for OCL differentiation. Tumor necrosis factor-related apoptosis inducing ligand (TRAIL) has been shown to increase osteoclastogenesis. We hypothesize that TRAIL may play an important role in μXg enhanced OCL differentiation. In this study, we identified by RT profiler PCR array screening that μXg induces high levels of TRAIL expression in murine preosteoclast cells in the absence of RANKL stimulation compared to ground based (Xg) cultures. We further identified that μXg elevated the adaptor protein TRAF-6 and fusion genes OC-STAMP and DC-STAMP expression in preosteoclast cells. Interestingly, neutralizing antibody against TRAIL significantly reduced μXg induced OCL formation. We further identified that over-expression of pTRAIL in RAW 264.7 cells enhanced OCL differentiation. These results indicate that TRAIL signaling plays an important role in the μXg increased OCL differentiation. Therefore, inhibition of TRAIL expression could be an effective countermeasure for μXg induced bone loss.

Weightlessness under microgravity (μXg) condition causes reduction in bone mineral density (BMD)^1^ which remains a major challenge for astronauts during long-term space mission^2^,^3^. Astronauts experience a total bone loss around 0–3% whereas bone loss in weight bearing bones such as spine and hip is about 0–20% depending upon their space flight exercise program, nutritional status and drug therapy under microgravity (μXg) conditions^4^,^5^. During spaceflight, lumbar spine, pelvis and proximal femur of the skeleton losses bone mineral density about 2–3% per month with an increase in urine excretion of calcium and hydroxyproline^6^,^7^,^8^. In addition, all the astronauts from long-term spaceflights have been recorded with T- or Z-scores less than −2.0 even after 10–15% BMD loss^9^. Also, fifteen days of space-flown (STS-131 space shuttle mission) mice have demonstrated 2 to 24% bone loss per month^10^. The morphological changes observed in astronaut’s bones after space flight also resembles osteoporotic patients^11^,^12^. Nutritional supplementation and exercise have been astronauts’ daily tasks^13^; however, bone loss remains a major challenge in space. Therefore, bone loss is a serious concern for long-term inhabitants of the space station and for space exploration. Recently, it has been reported that the combination of the advanced resistive exercise device (ARED) and bisphosphonates improved the weight bearing bone mineral density^14^,^15^. Although, mechano-stimulation influence bone strength and structure, the changes are irreversible with a poor recovery rate under microgravity^12^,^16^,^17^.

Osteoclast (OCL) is hematopoietic origin and differentiated into multinucleated cell which resorb bone^18^. The TNF family member receptor activator for nuclear factor κB ligand (RANKL) is critical for OCL precursor differentiation to form multinucleated OCL. RANKL interaction with RANK receptor activates various signaling cascades during OCL differentiation/activity^19^. Further, simulated μXg using the National Aeronautics and Space Administration (NASA) developed rotary cell culture system (RCCS) and hind-limb suspension of unloading in a murine model indicated the impact of μXg on bone cells^20^. Previously, it has been shown that osteopenia in the immobilized rat hind-limb model is associated with increased bone resorption and decreased bone formation^21^. Similarly, isolated fetal mouse long bones under near weightlessness conditions demonstrated decreased mineralization and increased calcium release^22^. Also, bone forming activity of osteoblast cells is decreased under μXg conditions^23^,^24^. Microgravity results in the uncoupling of bone remodeling between formation and resorption which could account for bone loss^25^,^26^. In addition, intact limb bones of the newts on board of a biosatellite Cosmos-2229 demonstrated elevated calcium content and histologic analysis revealed OCL activation on endosteal surface of long bones on 20^th^ day^27^. Studies during the FOTON-3 mission identified μXg directly target genes associated with OCL formation^28^. Several studies have been identified that μXg increases OCL bone resorptive activity^5^,^28^,^29^. Enhanced OCL differentiation under μXg has been shown with increase in collagen telopeptide levels in media samples retrieved from space^28^. Simulated μXg directly affects cell survival and differentiation of human preosteoclasts^30^. In addition, μXg aggravates RANKL-induced reactive oxygen species and increased OCL differentiation^31^. Evidence also suggests that NF-κB1 is involved with a rapid reduction of bone mass in space^32^. Also, it has been shown that OCLs were activated when the medaka fish were reared for 56 days at the international space station and the whole transcriptome analysis indicated fkbp5 and ddit4 genes were strongly up-regulated in the flight group^33^. Recently, we used the RCCS to simulate μXg and showed increased osteoclastogenesis. Also, microarray analysis of gene expression profiling in modeled μXg revealed significant increase in expression of critical molecules such as cytokines/growth factors, proteases and signaling proteins, which may play an important role in enhanced OCL differentiation/function^34^,^35^. In addition, gravity regulates RANKL and OPG expression in bone marrow stromal cells in the bone microenvironment^36^,^37^. However, the mechanisms of μXg induced bone loss and rational approaches to improve bone strength under weightlessness conditions are yet to be established.

Tumor necrosis factor-related apoptosis inducing ligand (TNSF10/Apo2L/TRAIL) is a member of the TNF superfamily^38^ expressed either as a type II transmembrane protein or as a soluble protein detectable in different biological fluids under physiological conditions^39^. TRAIL has been shown to induce apoptosis in malignant cells and at low doses promotes proliferation of normal mammalian cells^40^. In murine cells, TRAIL functions through DR5 which contain a death domain motif and also binds to DcR1, DcR2S (secreted form) and DcR2L (transmembrane form) receptors which lack of a death domain^41^,^42^,^43^. Also, TRAIL binds weakly with osteoprotegerin (OPG)^38^. It has been demonstrated that TRAIL induces OCL differentiation in pathological conditions^44^. Further, TRAIL activates TRAF6 dependent signaling pathway to increase OCL formation^45^. However, the role of TRAIL in microgravity included OCL formation is unknown.

In this study, we used the NASA developed ground-based RCCS to simulate μXg conditions and demonstrated high levels of TRAIL expression in preosteoclast cells in the absence of RANKL stimulation. We further identified that inhibition of TRAIL by a neutralizing antibody suppressed μXg induced OCL formation.

## Results

### Microgravity modulation of TRAIL/receptors expression in preosteoclast cells

**Figure 1:**
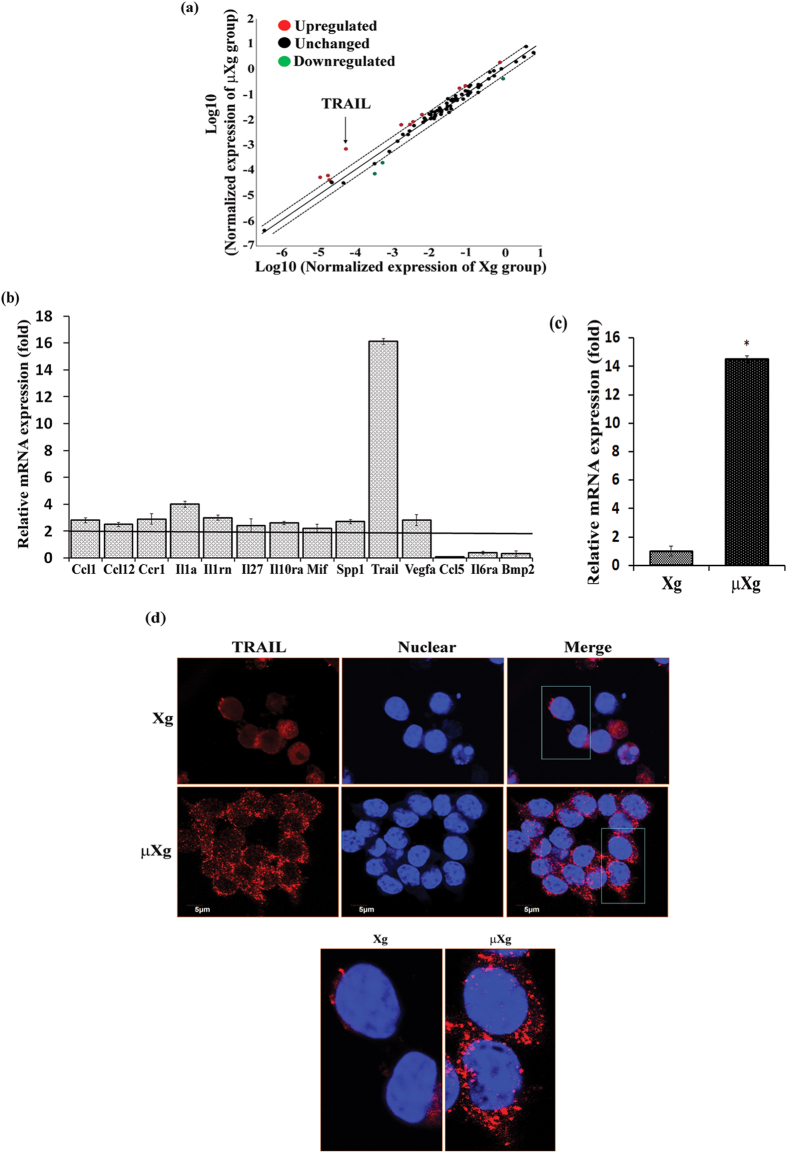
RT^2^ Profiler™ PCR Array screening and TRAIL expression in preosteoclast cells. RAW 264.7 cells were cultured as ground based control (Xg) or under μXg for 24 h. Total RNA was isolated from these cells were screened for RT Profiler array of 84 cytokine/growth factors mRNA expression using real-time PCR. The Array data were normalized with housekeeping gene panel B2M, HPRT1, RPL13A, GAPDH & ACTB and differential gene expression represented as (**a**) Scatter plot analysis (Red—upregulated; Black—unchanged; Green—downregulated); (**b**) bar graph showing specific gene expression changes. (**c**) Mouse bone marrow derived non-adherent cells were subjected to Xg or μXg for 24 h. Total RNA isolated from these groups was further analyzed for TRAIL mRNA expression using real-time RT-PCR. The mRNA expression was normalized with respect to β-actin amplification. Each bar represents the mean ± SD of three independent experiments. *Significant (P < 0.05) difference when compared to Xg. (**d**) Confocal microscopy analysis for TRAIL expression in Xg or μXg subjected preosteoclast cells. Nuclear staining was performed with DRAQ5 and TRAIL expression was visualized by IX81 confocal microscope. The boxed areas are shown digitally zoomed at higher magnification (63×) in the lower panel.

**Figure 2:**
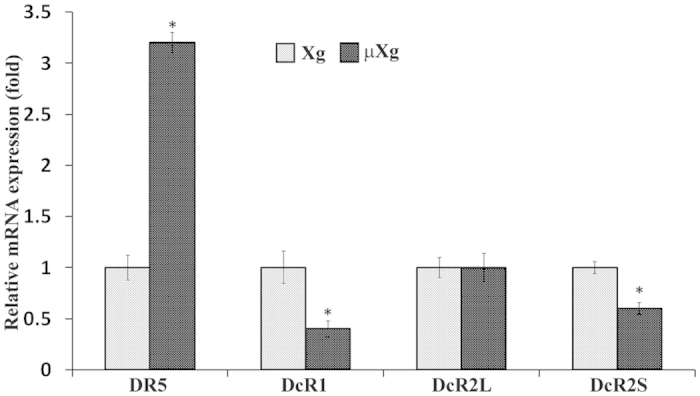
Differential TRAIL receptors expression under μXg. RAW 264.7 cells were cultured as ground based control (Xg) or μXg for 24 h and total RNA isolated were subjected to TRAIL receptors, DR5, DcR1, DcR2L and DcR2S mRNA expression using real-time RT-PCR. The mRNA expression was normalized with respect to β-actin mRNA amplification. Each bar represents the mean ± SD of three independent experiments. *Significant (P < 0.05) difference when compared to Xg.

Although, we previously demonstrated that simulated μXg by RCCS enhances OCL formation in mouse bone marrow cultures^35^, the molecular mechanisms involved are unclear. To further identify the μXg modulation of factors that enhances osteoclastogenesis, we subjected a homogeneous population of OCL progenitors RAW 264.7 cells to μXg in RCCS for optimal time point 24 h and total RNA isolated from these cells was utilized to screen RT Profiler array for 84 cytokine/growth factors by quantitative PCR analysis. The web portal analysis of array screening data for differential gene expression is shown as scatter plot (Fig. 1a). Various gene expression changes observed under μXg conditions is shown in Fig. 1b. We thus identified μXg upregulation of TRAIL expression in preosteoclast cells. Real-time RT-PCR analysis further confirmed μXg elevated (14.5-fold) TRAIL mRNA expression in mouse bone marrow derived preosteoclast cells (Fig. 1c). In addition, we performed confocal microscopy analysis for TRAIL expression in preosteoclast cells under μXg conditions. We identified that μXg strongly enhanced TRAIL expression in RAW 264.7 cells compared to ground based control (Xg) cultures (Fig. 1d). These results suggest that μXg directly stimulates TRAIL expression in preosteoclast cells. Microgravity regulation of TRAIL receptor expression in murine preosteoclast cells is unknown. Therefore, we further examined the μXg modulation of TRAIL receptor expression in RAW 264.7 cells. Cells subjected to μXg for 24 h and total RNA isolated was analyzed by real-time RT-PCR for TRAIL receptor mRNA expression. As shown in Fig. 2, μXg increased DR5 (3-fold) mRNA expression, whereas DcR1 and DcR2S expression levels were decreased when compared to cells cultured under normal Xg. However, no change was observed in DcR2L expression in these cells. These results indicate that μXg differentially regulates TRAIL receptors expression and that DR5 receptors expression is significantly upregulated in preosteoclast cells.

### Microgravity stimulates TRAF6 adaptor protein and fusion gene expression in preosteoclast cells

**Figure 3:**
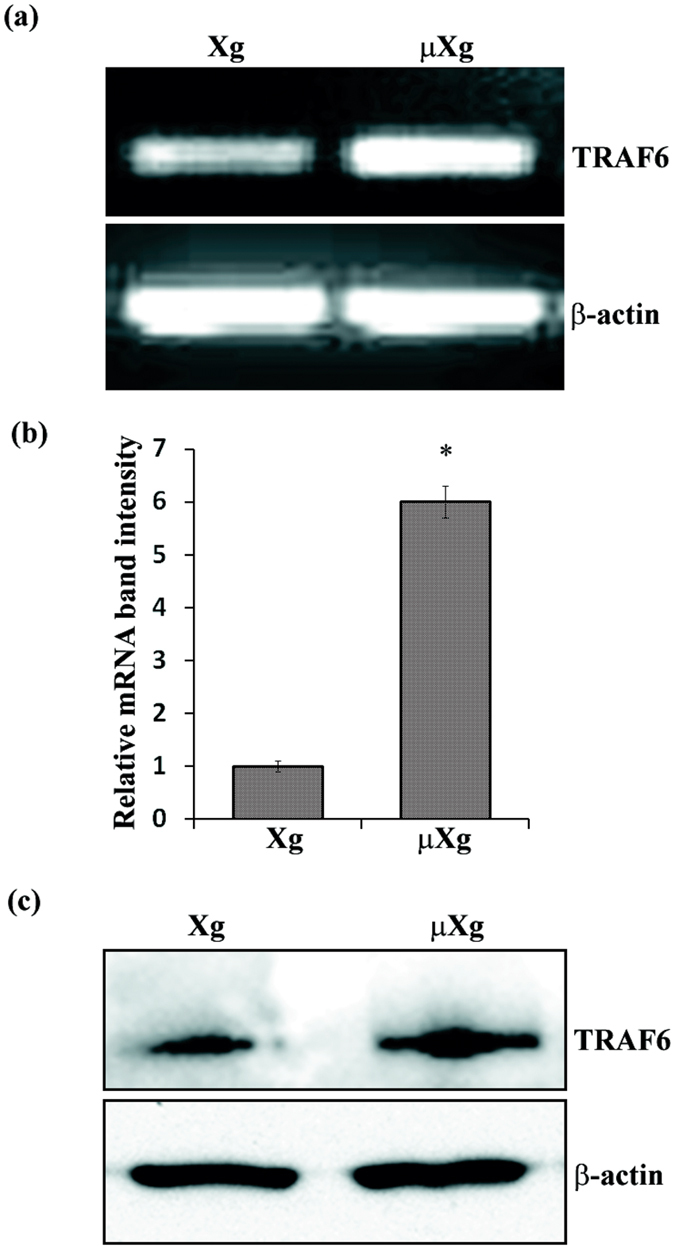
Microgravity stimulates TRAF6 expression in preosteoclast cells. (**a**) Mouse bone marrow derived non-adherent cells were cultured in ground (Xg) or subjected to μXg for 24 h. Total RNA isolated from these cells was analyzed for TRAF6 mRNA expression using RT-PCR and (**b**) the band intensity was measured by ImageJ. The relative band intensity was normalized by β-actin amplification in these cells. Each bar represents the mean ± SD of three independent experiments. *Significant (P < 0.05) difference when compared to Xg. (**c**) Western blot analysis of TRAF6 expression in mouse bone marrow derived preosteoclast cells under μXg conditions. Cells were cultured in Xg or μXg for 24 h and total cell lysates were analyzed for TRAF6 expression. β-actin expression serves as control.

**Figure 4:**
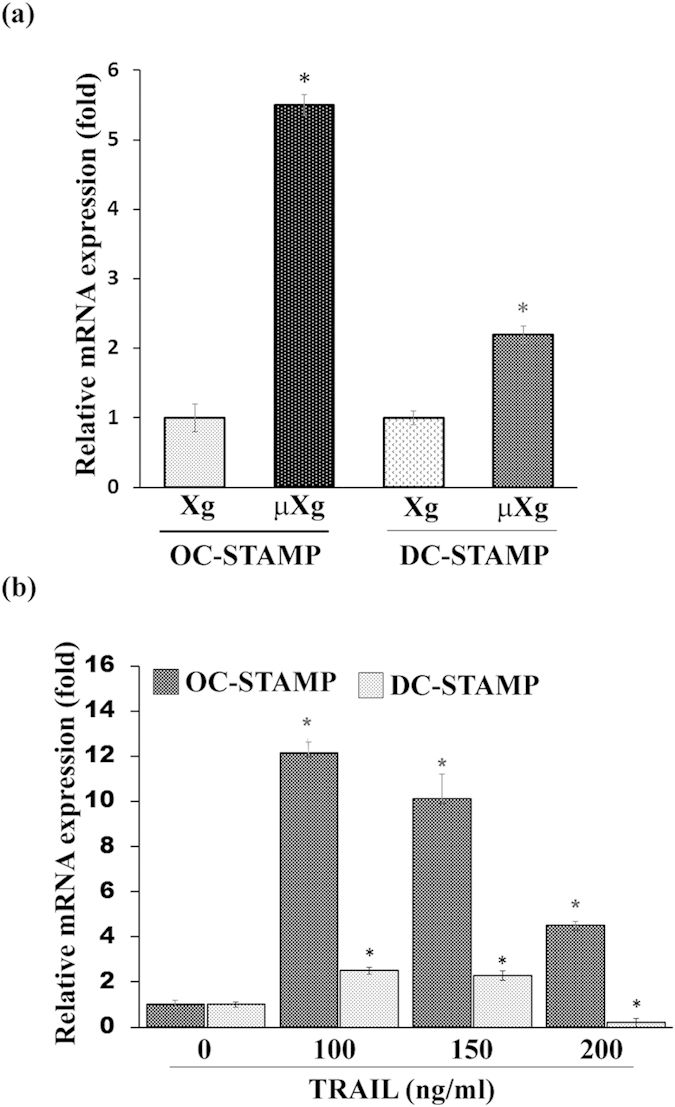
Microgravity and TRAIL stimulates fusion gene expression in preosteoclast cells. (**a**) Total RNA isolated from preosteoclast cells cultured in Xg or μXg for 24 h was analyzed for OC-STAMP and DC-STAMP mRNA expression using real-time RT-PCR. (**b**) Real-time RT-PCR analysis of OC-STAMP and DC-STAMP mRNA expression in preosteoclast cells treated with different concentration of TRAIL (0–200 ng/ml). The mRNA expression was normalized with respect to β-actin amplification. Each bar represents the mean ± SD of three independent experiments. *Significant (P < 0.05) difference when compared to Xg or without TRAIL treatment.

Both RANKL and TRAIL has been shown to signals through TRAF6 adaptor protein in preosteoclast cells^45^,^46^. Therefore, we examined the μXg regulation of TRAF6 expression in mouse bone marrow derived preosteoclast cells. As shown in Fig. 3a,b, the TRAF6 mRNA expression was significantly increased (6-fold) in μXg subjected mouse bone marrow derived non-adherent cells compared to ground based control cells. Further, western blot analysis of total cell lysates obtained from these cells demonstrated a significant increase (4.5-fold) in the levels of TRAF6 protein expression in the absence of RANKL stimulation (Fig. 3c). Dendritic cell-specific transmembrane protein (DC-STAMP) and osteoclast-stimulatory transmembrane protein (OC-STAMP) have been identified as essential factors for preosteoclast fusion to form multinucleated giant cells^47^,^48^. Therefore, we further examined the μXg effect of OC-STAMP and DC-STAMP expression in preosteoclast cells. Real-time RT-PCR analysis showed that μXg elevated OC-STAMP mRNA expression (5.5-fold), whereas DC-STAMP moderately increased (2.2-fold) in these cells (Fig. 4a). To test whether TRAIL regulate OC-STAMP and DC-STAMP expression, we treated the preosteoclast cells with different concentration of TRAIL (0–200 ng/ml) for 24 h. As shown in Fig. 4b, real-time RT-PCR analysis demonstrated that TRAIL treatment at 100 ng/ml concentration significantly increased OC-STAMP (12-fold) and DC-STAMP (2.5-fold) expression. These results suggest that μXg modulation of TRAF6 adaptor protein and fusion gene mRNA expression may play an important role in TRAIL induced OCL differentiation under μXg conditions.

### Microgravity but not RANKL induces TRAIL expression in preosteoclast cells

**Figure 5:**
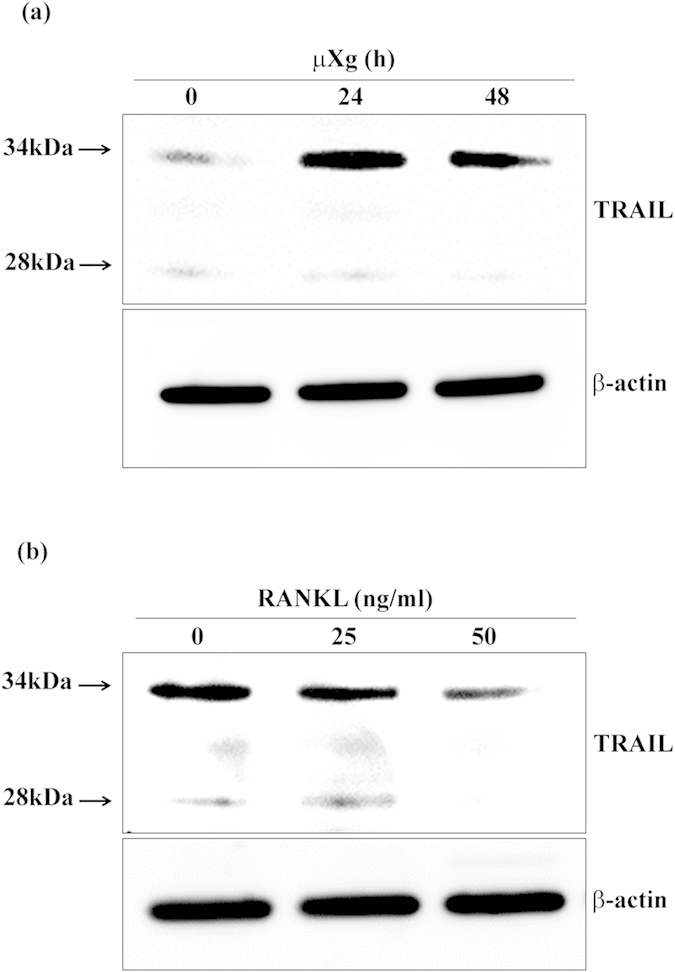
Effect of μXg and RANKL on TRAIL expression. (**a**) Mouse bone marrow derived non-adherent cells were cultured under μXg for different time-points (0–48 h) and total cell lysates (50 μg of protein) were analyzed for TRAIL expression by western blot. (**b**) Western blot analysis of TRAIL expression in RANKL treated preosteoclast cells. Mouse bone marrow derived non-adherent cells were treated with different concentration of RANKL (0–50 ng/ml) for 24 h and total cell lysates (100 μg of protein) were analyzed for TRAIL expression. β-actin expression serves as control.

We further examined μXg modulation of TRAIL expression in preosteoclast cells at variable time points. Western blot analysis of total cell lysates obtained from mouse bone marrow derived non-adherent cells cultured under μXg condition for different time points (0–48 h) revealed 8-fold and 6-fold increase in the level of TRAIL protein expression at 24 and 48 h, respectively (Fig. 5a). Since RANKL is critical for OCL differentiation in the bone microenvironment, we next examined the RANKL regulation of TRAIL expression. RANKL treatment at variable doses (0–50 ng/ml) for 24 h had a dose-dependent decrease in TRAIL expression in mouse bone marrow derived preosteoclast cells (Fig. 5b). These results indicate that μXg but not RANKL induce TRAIL expression in preosteoclast cells to enhance OCL differentiation.

### TRAIL participation in μXg enhanced osteoclast (OCL) formation

**Figure 6:**
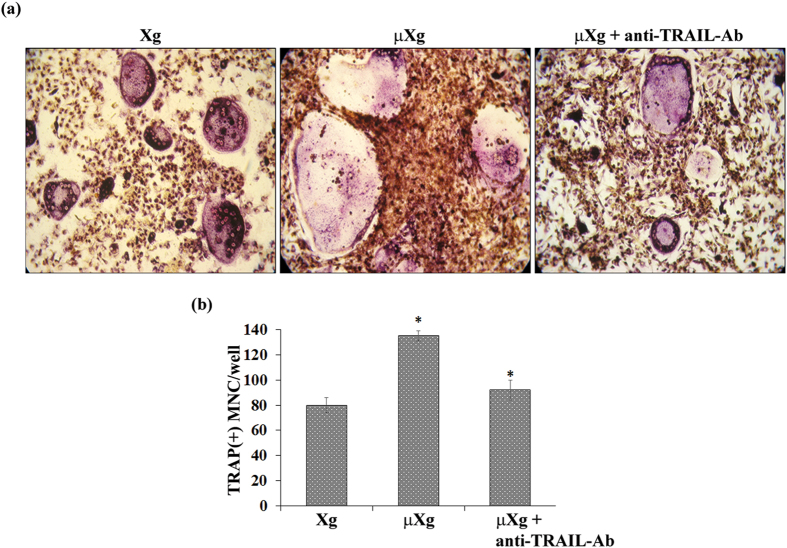
Neutralizing antibody against TRAIL inhibits μXg induced osteoclast (OCL) differentiation. (**a**) Mouse bone marrow non-adherent cells were cultured as ground based control (Xg) or subjected to μXg for 24 h and treated with RANKL (50 ng/ml) and M-CSF (10 ng/ml) for 7 days in the presence and absence of anti-TRAIL antibody (5 μg/ml) for OCL formation. (**b**) TRAP-positive multinucleated OCLs formed at the end of the culture period were scored under microscope. *Significant (P < 0.05) difference when compared to Xg.

**Figure 7:**
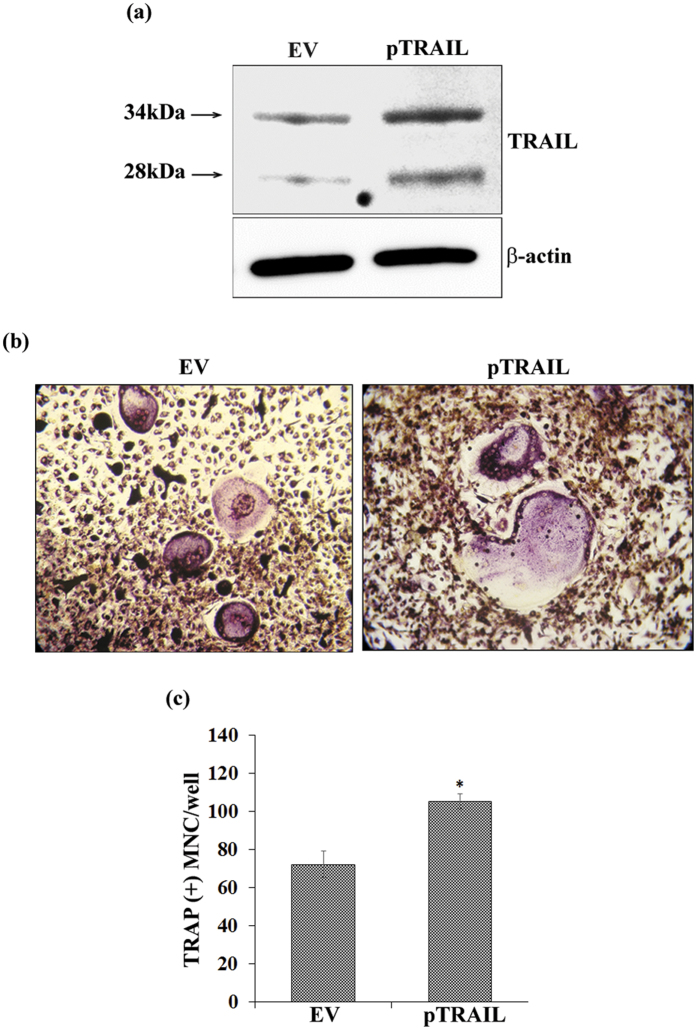
Over-expression of TRAIL increases osteoclastogenesis. (**a**) Ground based control RAW 264.7 cells were transfected with empty vector (EV) or TRAIL expression vector (pTRAIL) and over-expression of TRAIL was confirmed after 48 h by western blot. β-actin expression served as control; (**b**) cultured for OCL formation. (**c**) TRAP-positive multinucleated OCLs formed at the end of the culture period were scored under microscope. *Significant (P < 0.05) difference when compared to EV.

We next examined whether μXg induced TRAIL expression involved in enhanced OCL differentiation. Mouse bone marrow derived non-adherent cells were subjected to μXg for 24 h and treated with RANKL and M-CSF for 7 days in the presence and absence of anti-TRAIL antibody (5 μg/ml). Tartrate-resistant acid phosphatase (TRAP) positive multinucleated cells (MNC) formed in these cultures were scored. Interestingly, cells treated with a neutralizing antibody against TRAIL inhibit μXg induced OCL differentiation (Fig. 6a,b). To further confirm the role of TRAIL in OCL formation, RAW 264.7 cells were transiently transfected with empty vector (EV) or TRAIL expression vectors (pTRAIL) as described in Methods for 2 days. Western blot analysis of total cell lysates obtained from these cells showed a 7-fold increase in the levels of TRAIL expression compared to EV (Fig. 7a). Furthermore, TRAIL over-expression in preosteoclast cells showed a significant increase in TRAP positive MNC formation (Fig. 7b,c). These results indicate that increased levels of TRAIL expression in preosteoclast cells enhance OCL differentiation under μXg conditions.

## Discussion

Microgravity (μXg) in space could alter normal bone homeostasis. Osteoclast (OCL) being the primary bone resorbing cell, it is important to unravel the molecular mechanisms underlying the μXg control of OCL formation. Previously, we and others have reported that microgravity induce transcription factors which regulate gene expression^34^,^49^,^50^. This study identifies the increased levels of TRAIL expression in preosteoclast cells subjected to simulated μXg conditions in the absence of RANKL stimulation suggest a direct effect of microgravity on TRAIL expression at gene transcription level. TRAIL isoforms (α, β and γ) have been identified and implicated in cellular growth/differentiation^40^. However, in this study we show microgravity upregulation of TRAIL isoform (34 kDa) predominantly in preosteoclast cells and that RANKL downregulates TRAIL expression. TRAIL has been reported to induces apoptosis in malignant cells and promote proliferation of normal cells^40^. Therefore, it is possible that enhanced levels of OCL formation under μXg conditions could be due to increased numbers of preosteoclast cells. TRAIL is expressed either as a type II transmembrane or soluble protein^39^ and has been localized in both cytoplasm and membrane^40^,^51^. Our confocal microscopy analysis revealed μXg enhanced TRAIL expression in preosteoclast cells and consistent with cellular localization as reported. Further, this study demonstrated that μXg increases DR5 and decrease DcR1, DcR2s TRAIL receptor expression suggests that DR5 could play an important role in TRAIL signaling in preosteoclast cells. It has been reported that binding of TRAIL to DR5 has no physiologic relevance^52^. However, μXg modulation of DR5 expression suggests that TRAIL signaling through DR5 receptors may have pathologic significance under low gravity conditions. Our results favor previous findings that TRAIL induces osteoclast differentiation via direct engagement with the TRAIL death receptor through a signaling pathway distinct from apoptosis^53^. Since the expression of other TRAIL receptors such as DcR1 and DcR2L is also detected in preosteoclast cells, we cannot rule out the participation of these receptors in osteoclast differentiation. This study identifies that μXg also enhanced the levels of TRAF6 expression in preosteoclast cells without RANKL stimulation. TRAF6 is an adaptor protein critical for RANKL-RANK receptor signaling essential for OCL differentiation. Recent evidence also indicates that TRAF6 mediates TRAIL signaling to increase OCL differentiation^45^. Therefore, it is possible that μXg induction of TRAIL may signals through TRAF6 dependent pathway to promote OCL formation under low gravity. We recently demonstrated that μXg induction of autophagy modulates OCL formation^35^. It has been shown that TRAIL induces cytoprotective autophagy in un-transformed cells^54^. Therefore, TRAIL signaling may promote autophagy process for survival of OCL progenitor cells under stress in low gravity conditions.

It has been demonstrated that inhibition of PI 3-kinase signaling results in induction of TRAIL expression play a role in intestinal cell homeostasis. Also, NFATc1 activation through suppression of Sp1 transcription factor increased TRAIL expression in human intestinal cells^55^,^56^. We have recently demonstrated that RANKL induction by TRAIL via STAT-6 dependent pathway in bone marrow stroma/preosteoblast cells^57^. TRAIL receptor signaling has been implicated in cytokine production and NF-kB activation in macrophages^58^. Therefore, understanding the μXg regulation of signaling pathways and downstream effectors that would modulate TRAIL expression in preosteoclast cells would provide important insights to develop countermeasures for bone loss under microgravity.

As evident from the results using the neutralizing antibody against TRAIL, we showed that TRAIL participates in μXg enhanced OCL differentiation. Further, over-expression of TRAIL in preosteoclast cells enhanced OCL differentiation in the presence of RANKL. This study also demonstrated that μXg elevated the fusion gene expression such as OC-STAMP and DC-STAMP expression essential for OCL formation. In fact, our results indicated that OC-STAMP expression is highly pronounced compared to DC-STAMP in preosteoclast cells under μXg conditions. Furthermore, TRAIL modulates fusion gene expression suggest a potential role for TRAIL in μXg induced OCL formation. In contrast, we observe μXg had no significant effect (data not shown) on other molecules such as Pim-1, an intracellular signaling molecule suggested to plays a role in OCL formation^59^. Therefore, it is likely that μXg increased TRAIL expression in preosteoclast cells promotes osteoclastogenesis and could be a potential therapeutic target to control bone loss in space.

## Methods

### Rotary Cell Culture System (RCCS)

We used NASA developed RCCS (Synthecon Inc, TX) to simulate microgravity (μXg) conditions as we described^34^. RCCS is a horizontally rotated cell culture vessel with a silicone membrane which will allow oxygenation and co-localization of particles with different sedimentation rates^24^. Suspension of cells in the culture vessel will be uniform with a reduced fluid shear forces and three-dimensional (3D) spatial freedom. Mouse bone marrow-derived non-adherent cells or RAW 264.7 cells cultured in RCCS with α-MEM containing 10% fetal bovine serum (FBS) was rotated at 16 rpm for 24 or 48 h to simulate a microgravity (0.008 g) environment (μXg), in a humidified incubator at 37 °C with 5% CO_2_. Ground based cells cultured under normal gravity (Xg) served as control.

### RT^2^ Profiler™ PCR Array Screening

Total RNA was isolated from RAW 264.7 cells subjected to μXg or ground based control (Xg) conditions. The reverse transcription reaction was performed using poly-dT primer and moloney murine leukemia virus reverse transcriptase in a 25 μl reaction volume containing total RNA (2 μg), 1 × PCR buffer and 2 mM MgCl_2_, at 42 °C for 15 min followed by 95 °C for 5 min. The RT^2^ Profiler PCR Array (PAMM-011z SABiosciences, Valencia, CA) screening of 84 cytokine genes was performed by qPCR using IQ™ SYBR Green Supermix in an iCycler. Thermal cycling parameters were 95 °C for 10 min, followed by 40 cycles of amplifications at 95 °C for 15 s, 55 °C for 30 s, 72 °C for 30 s, and 72 °C for 5 min as the final elongation step. Relative levels of mRNA expression were normalized in all the samples with expression levels of housekeeping genes (GUSB, HPRT, HSP90AB1, GAPDH and β-actin) mRNA amplification. The data is analyzed using the GeneGlobe Data Analysis Center from web resource.

### Real-time RT-PCR

The quantitative real-time RT-PCR was performed using IQ^TM^ SYBR Green Supermix in an iCycler (iCycler iQ Single-color real-time-PCR detection system; Bio-Rad, Hercules, CA). The primer sequences used to amplify mRNA expression were as follows: β-actin 5′-TGA GAG GGA AAT CGT GCG TGA-3′ (sense) and 5′-AAG AAG GAA GGC TGG AAA AGA G-3′ (anti-sense); TRAIL 5′-CGG AGA AGC AAC TCA GCT TTA-3′ (sense) and 5′-GTT GAG AAA TGA ATG CCC TTT CC-3′ (anti-sense); DR5 5′-GAT CTG CCA GTC ATG CTC TAA C-3′ (sense) and 5′-CCT ATC CAG AGG CCT AGC TTA T-3′ (anti-sense); DcR1 5′-CTG AGT CAC TGG TTC CTC TTG-3′ (sense) and 5′-CGG GAC AGT TGA AGG AGT ATG-3′ (anti-sense); DcR2L 5′-ATG CAA CTC CAC AGC TAA CA-3 (sense) and 5′-TCA AAG GTG ATA ACA GTA GGA ACA-3′ (anti-sense); DcR2S 5′-CCA TAC TCA AGG ACA ATG TGA GA-3 (sense) and 5′-GAT CTT TAT CAC AGG TGG AGC A-3′ (anti-sense); OC-STAMP 5′-ATG AGG ACC ATC AGG GCA GCC ACG-3 (sense) and 5′-GGA GAA GCT GGG TCA GTA GTT CGT-3′ (anti-sense); DC-STAMP 5′-TCC TCC ATG AAC AAA CAG TTC CAA-3 (sense) and 5′-AGA CGT GGT TTA GGA ATG CAG CTC-3′ (anti-sense). Thermal cycling parameters were 95 °C for 4 min, followed by 38 cycles of amplifications at 95 °C for 1 min, 55 °C for 1 min, 72 °C for 2 min, and 72 °C for 10 min as the final elongation step. The melt curve analysis was performed from 59–95 °C with 0.5 °C increments. The specificities of PCR amplifications were assessed from the melt curves to confirm the presence of gene specific peaks. Relative levels of mRNA expression were normalized in all the samples analyzed with respect to the levels of β-actin mRNA amplification.

### Osteoclast (OCL) Culture

Bone marrow was flushed from long bones of 6–8 week-old mice (C57BL/6) using α-MEM. Cells were pelleted at 1500 rpm for 7 min at room temperature and plated in α-MEM with 10% FBS supplemented with M-CSF (10 ng/ml) and cultured overnight. Non-adherent mouse bone marrow cells (1.5 × 10^6^/ml) subjected to μXg in RCCS for 24 h were cultured in a 96 well plate for 7 days with M-CSF (10 ng/ml), RANKL (75 ng/ml) (R&D Systems Inc., Minneapolis, MN) in the presence and absence of anti-TRAIL antibody (5 μg/ml). Cells were fixed with 2% glutaraldehyde in phosphate buffered saline (PBS) and stained for TRAP activity using a histochemical kit (Sigma Chemical Co., St. Louis, MO). TRAP positive multinucleated cells (MNC) containing three or more nuclei were scored as OCLs under a light microscope as described^35^. The animal procedures were approved by the Institutional Animal Care and Use Committee of the Medical University of South Carolina and carried out in accordance with the approved guidelines.

### Confocal Microscopy

Ground based control (Xg) and μXg subjected RAW 264.7 cells (1 × 10^4^/well) were seeded onto 22 mm coverslips in 6-well plates. After 24 h, cells were then fixed with 4% paraformaldehyde in PBS for 10 min at room temperature. Cells were permeabilized with 0.1% Triton X-100 for 5 min and blocked for 1 h with PBS containing 10% donkey serum. Cells were incubated with primary antibody against TRAIL (1:100 dilution) in PBS overnight at 4 °C. After extensive washings with PBS, cells were incubated with Alexa 568-conjugated anti-rabbit IgG. Nuclear staining was performed with DRAQ5 and localization of TRAIL was visualized by IX81 confocal microscope (Olympus, IX81).

### TRAIL Over-expression

RAW 264.7 cells were seeded (5 × 10^5^ cells/well) in 6-well plates and supplemented with MEM containing 10% FBS. A day after seeding, cells were transiently transfected with 4 ng/ml of TRAIL expression construct (pCR2.1 mouse TRAIL B1/pTRAIL) or empty vector (EV) by lipofectamine. The cells were cultured for 48 h period and the over-expression of TRAIL was confirmed by western blot analysis. Further, the transfected RAW 264.7 cells were cultured in the presence of M-CSF and RANKL for 5 days to form OCLs as described above.

### Western Blot

Mouse bone marrow derived non-adherent or RAW 264.7 cells supplemented with α-MEM containing 10% FBS were subjected to μXg for different time points (0–48 h) or transfected with pTRAIL or stimulated with various concentration of RANKL (50 ng/ml) for 24 h. Total cell lysates were prepared in a buffer containing 20  mM Tris–HCl at pH 7.4, 1% Triton X-100, 1 mM EDTA, 1.5  mM MgCl2; 10% glycerol, 150 mM NaCl, 0.1  mM Na3VO4 and 1 × protease inhibitor cocktail. The protein content of the samples was measured using the Bradford protein assay reagent (Bio-Rad, Hercules, CA). Samples were then subjected to SDS–PAGE using 4–15% Tris–HCl gradient gels and blot transferred on to a PVDF membrane, immunoblotted with anti-TRAIL (Abcam, Cambridge, MA), anti-TRAF6 (Santa Cruz Biotechnology Inc., CA) and anti-β-actin (R&D Systems Inc., Minneapolis, MN) antibodies. The bands were detected using the enhanced chemiluminescence detection system (Pierce, Rockford, IL) and band intensity was quantified by NIH ImageJ.

### Statistical Analysis

Results are presented as mean ± SD for three independent experiments and were compared by Student t-test. Values were considered significantly different for p < 0.05.

## Additional Information

**How to cite this article**: Sambandam, Y. *et al.* Microgravity Induction of TRAIL Expression in Preosteoclast Cells Enhances Osteoclast Differentiation. *Sci. Rep.* **6**, 25143; doi: 10.1038/srep25143 (2016).
